# 1783. Appropriateness of antibiotics use for patients with asymptomatic bacteriuria or urinary tract infection: A retrospective observational multicenter study in Korea

**DOI:** 10.1093/ofid/ofac492.1413

**Published:** 2022-12-15

**Authors:** Jongtak Jung, Bongyoung Kim, Dong Youn Kim, Mi Suk Lee, Se Yoon Park, Tae Hyong Kim, Myung Jin Lee, Ji Young Park, Hee Bum Jo, Woo Joo Lee, Jin Yong Kim, Song Mi Moon, Kyoung-Ho Song, Jeong Su Park, Eu Suk Kim, Min Hyung Kim, Yoon Soo Park, Yee Gyung Kwak, Ji-Yeon Kim, Jeanno Park, Young Keun Kim, Hye Won Jeong, Sun Hee Park, Joon Hwan An, JaeHoon Lee, Kyung-Hwa Park, Sohyun Bae, Hyun-Ha Chang, Si-Ho Kim, Deog-Hyeon Son, HoJin Lee, Chisook Moon, Sang Taek Heo, Jaehun Jung, Hong Bin Kim

**Affiliations:** Soonchunhyang University Seoul Hospital, Seoul, Seoul-t'ukpyolsi, Republic of Korea; Department of Internal Medicine, Hanyang University College of Medicine, Seongdong-gu, Seoul-t'ukpyolsi, Republic of Korea; Division of Infectious Diseases, Department of Internal Medicine, Kyung Hee University Hospital, Kyung Hee University School of Medicine, Seoul, Republic of Korea, Seoul, Seoul-t'ukpyolsi, Republic of Korea; Division of Infectious Diseases, Department of Internal Medicine, Kyung Hee University Hospital, Kyung Hee University School of Medicine, Seoul, Seoul-t'ukpyolsi, Republic of Korea; Division of Infectious Diseases, Department of Internal Medicine, Soonchunhyang University Seoul Hospital, Seoul, Seoul-t'ukpyolsi, Republic of Korea; Division of Infectious Diseases, Department of Internal Medicine, Soonchunhyang University Seoul Hospital, Seoul, Seoul-t'ukpyolsi, Republic of Korea; Department of Internal Medicine, Inje University Sanggye-Paik Hospital, Seoul, South Korea, Seoul, Seoul-t'ukpyolsi, Republic of Korea; Department of Pediatrics, Chung-Ang University Hospital, Seoul, Korea, Seoul, Seoul-t'ukpyolsi, Republic of Korea; Division of Infectious Diseases, Department of Internal Medicine, Incheon Sejong Hospital, Incheon, Korea, Seoul, Seoul-t'ukpyolsi, Republic of Korea; Department of Internal Medicine, Hallym Hospital, Incheon, South Korea., Seoul, Seoul-t'ukpyolsi, Republic of Korea; Department of Internal Medicine, Incheon Medical Center, Incheon, Korea., Seoul, Seoul-t'ukpyolsi, Republic of Korea; Seoul National University College of Medicine, Seoungnam-si, Kyonggi-do, Republic of Korea; Seoul National University College of Medicine, Seoungnam-si, Kyonggi-do, Republic of Korea; Department of Laboratory Medicine, Seoul National University Bundang Hospital, Seoul, Seoul-t'ukpyolsi, Republic of Korea; Seoul National University College of Medicine, Seoungnam-si, Kyonggi-do, Republic of Korea; Division of Infectious Diseases, Department of Internal Medicine, Yongin Severance Hospital, Yonsei University College of Medicine, Seoul, Seoul-t'ukpyolsi, Republic of Korea; Department of Internal Medicine, Division of Infectious disease, Yongin Severance Hospital, Yonsei University College of Medicine, Yongin, Kyonggi-do, Republic of Korea; Inje University Ilsan Paik Hospital, Seoul, Seoul-t'ukpyolsi, Republic of Korea; Division of Infectious Disease, Department of Medicine, Seongnam Citizens Medical Center, Seongnam, Korea., Seongnam city, Kyonggi-do, Republic of Korea; Palliative Care and Hospice Center, Bobath Memorial Hospital, Bundang, Seongnam, Republic of Korea., Seoul, Seoul-t'ukpyolsi, Republic of Korea; Yonsei University Wonju College of Medicine, Wonju, Kangwon-do, Republic of Korea; Department of Internal Medicine, Chungbuk National University Hospital, Cheongju, Republic of Korea; Department of Internal Medicine, Chungbuk National University College of Medicine, Cheongju, Republic of Korea, Cheongju, Ch'ungch'ong-bukto, Republic of Korea; Division of Infectious Diseases, Department of Internal Medicine , The Catholic University of Korea, Seoul, Korea, Seoul, Seoul-t'ukpyolsi, Republic of Korea; Mokpo Hankook Hospital, Mokpo, Ch'ungch'ong-namdo, Republic of Korea; Department of Internal Medicine, Wonkwang University College of Medicine, Iksan, Korea., Iksan, Ch'ungch'ong-namdo, Republic of Korea; Chonnam National University Medical School, GwangJu, Kwangju-jikhalsi, Republic of Korea; Division of Infectious Diseases, Department of Internal Medicine, Kyungpook National University Hospital, School of Medicine, Kyungpook National University, Daegu, Republic of Korea, Taegu, Taegu-jikhalsi, Republic of Korea; Division of Infectious Diseases, Department of Internal Medicine, Kyungpook National University Hospital, School of Medicine, Kyungpook National University, Daegu, Republic of Korea, Taegu, Taegu-jikhalsi, Republic of Korea; Division of Infectious Diseases, Samsung Changwon Hospital, Sungkyunkwan University, Changwon, Korea., Changwon, Kyongsang-namdo, Republic of Korea; Eson Convalescent Hospital, Ulsan, Ulsan-gwangyoksi, Republic of Korea; Department of Infectious Diseases, Dong-A University Hospital, Pusan, Pusan-jikhalsi, Republic of Korea; Division of Infectious disease, Department of Internal Medicine, Busan Paik Hospital, College of Medicine, Inje University, Busan, Korea, Pusan, Pusan-jikhalsi, Republic of Korea; Department of Infectious Disease, Jeju National University School of Medicine, Jeju, South Korea, Jeju, Cheju-do, Republic of Korea; Gil Medical Centre, Gachon University College of Medicine, Incheon, Inch'on-jikhalsi, Republic of Korea; Seoul National University College of Medicine, Seoungnam-si, Kyonggi-do, Republic of Korea

## Abstract

**Background:**

Antibiotic resistance threatens public health worldwide, and inappropriate use of antibiotics is one of the main causes. Antibiotic use for asymptomatic bacteriuria (ABU) has been defined as “antibiotics never events”, and urinary tract infection (UTI) is one of the most common infectious diseases for which antibiotics are prescribed in Korea. To establish an effective antimicrobial stewardship strategy, a qualitative assessment of antibiotic use in actual clinical syndrome is necessary.

**Methods:**

Cases of positive urine cultures (≥10^5^ CFU/ml), performed in inpatient, outpatient, and emergency departments in April 2021 were screened in 26 hospitals located throughout Korea. Cases were classified into ABU, lower UTI, and upper UTI. The appropriateness of antibiotic use was retrospectively evaluated by infectious disease specialists using quality indicators based on the domestic clinical guideline for ABU and UTI.

**Figure 1.**
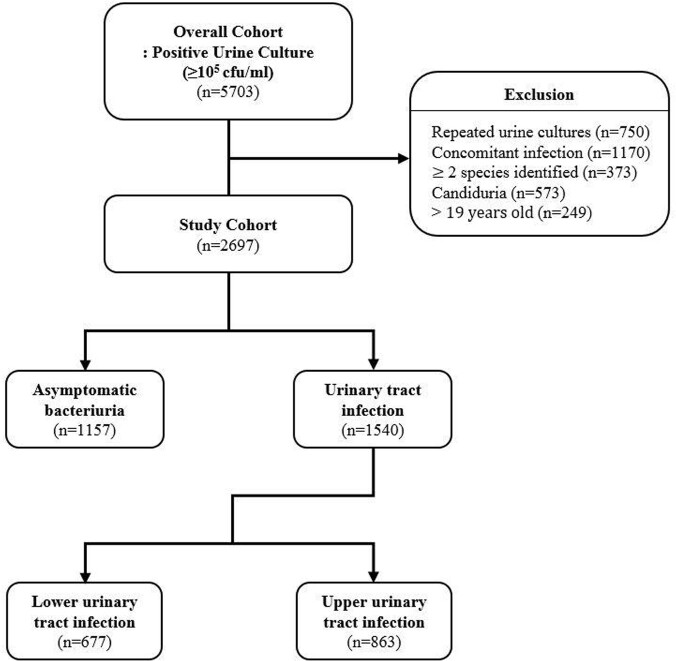
Study flow diagram

**Results:**

A total of 2697 cases of ABU or UTI were included. The appropriateness of antibiotic use was assessed in 1157 cases with asymptomatic bacteriuria, 677 and 863 cases with lower and upper UTI (Figure 1). Antibiotics were prescribed in 21.7% (251 of 1157) of ABU without appropriate indication. Of 66 ABU cases with appropriate indication in which prophylactic antibiotics were prescribed, the duration of antibiotics was adequate in only 34.8% (Table 1). For lower UTI, the appropriateness of empirical and definite antibiotics was 77.8% (527 of 677) and 68.0% (353 of 519). In terms of upper UTI, 86.3% (745 of 863) and 78.2% (583 of 746) was appropriate, respectively. The duration of antibiotics was adequate in 65.7% (421 of 641) of lower UTI and 77.9% (592 of 760) in upper UTI (Table 2, 3).

**Figure:**
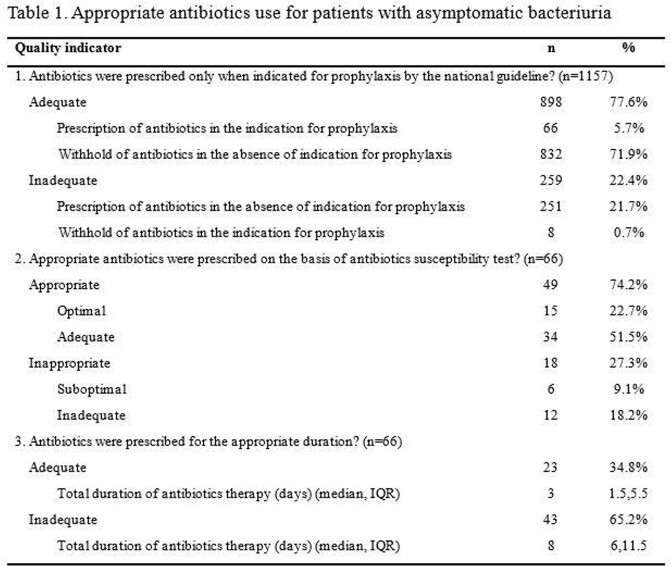

**Figure:**
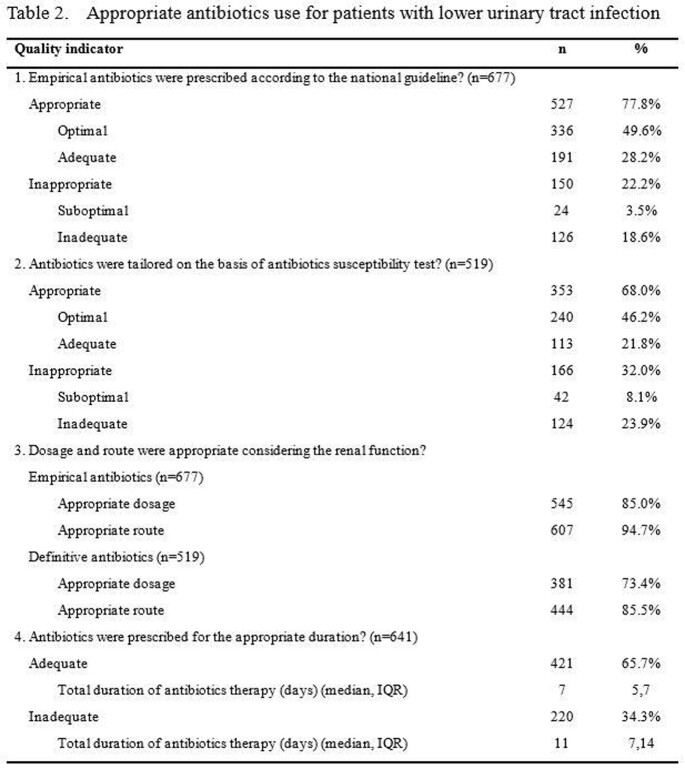

**Figure:**
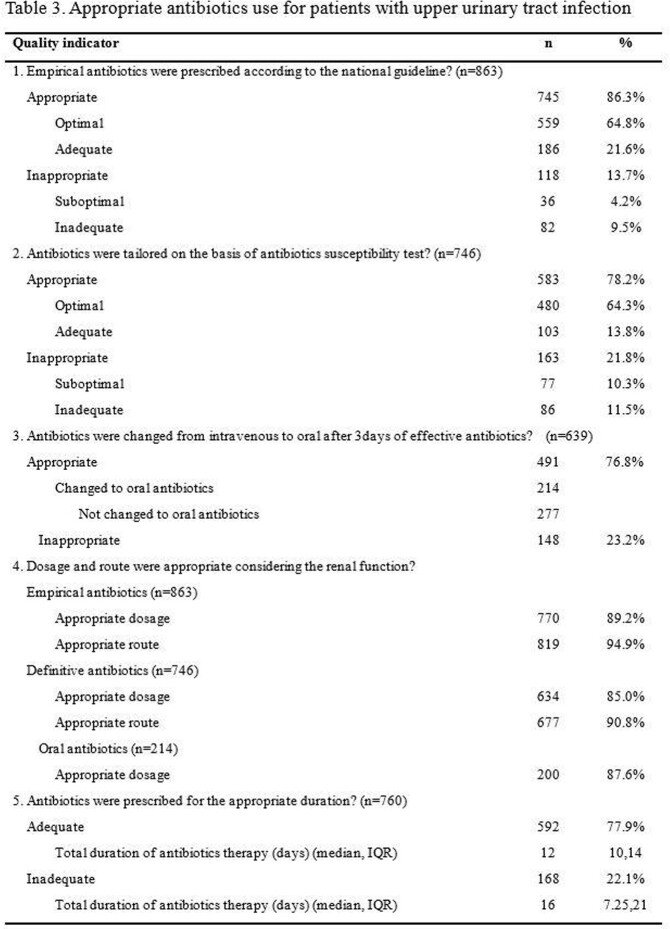

**Conclusion:**

This nationwide qualitative assessment of antibiotic use in ABU and UTI revealed that a significant proportion of antibiotics were prescribed inappropriately and, furthermore the duration of antibiotics was prolonged unnecessarily. Interventions for appropriate antibiotic use in ABU and UTI at the national level are required.

**Disclosures:**

**All Authors**: No reported disclosures.

